# Workflows for Rapid Functional Annotation of Diverse Arthropod Genomes

**DOI:** 10.3390/insects12080748

**Published:** 2021-08-19

**Authors:** Surya Saha, Amanda M. Cooksey, Anna K. Childers, Monica F. Poelchau, Fiona M. McCarthy

**Affiliations:** 1 Boyce Thompson Institute, 533 Tower Rd., Ithaca, NY 14853, USA; suryasaha@cornell.edu; 2 School of Animal and Comparative Biomedical Sciences, University of Arizona, 1117 E. Lowell St., Tucson, AZ 85721, USA; amcooksey@arizona.edu; 3 CyVerse, BioScience Research Laboratories, University of Arizona, 1230 N. Cherry Ave., Tucson, AZ 85721, USA; 4 Bee Research Laboratory, Beltsville Agricultural Research Center, Agricultural Research Service, USDA, 10300 Baltimore Ave., Beltsville, MD 20705, USA; anna.childers@usda.gov; 5 National Agricultural Library, Agricultural Research Service, USDA, 10301 Baltimore Ave., Beltsville, MD 20705, USA; monica.poelchau@usda.gov

**Keywords:** functional annotation, Gene Ontology, pathways, annotation, workflow, invertebrate

## Abstract

**Simple Summary:**

Genomic technologies are accumulating information about genes faster than ever before, and sequencing initiatives, such as the Earth BioGenome Project, i5k, and Ag100Pest Initiative, are expected to increase this rate of acquisition. However, if genomic sequencing is to be used for the improvement of human health, agriculture, and our understanding of biological systems, it is necessary to identify genes and understand how they contribute to biological outcomes. While there are several well-established workflows for assembling genomic sequences and identifying genes, understanding gene function is essential to create actionable knowledge. Moreover, this functional annotation process must be easily accessible and provide information at a genomic scale to keep up with new sequence data. We report a well-defined workflow for rapid functional annotation of whole proteomes to produce Gene Ontology and pathways information. We test this workflow on a diverse set of arthropod genomes and compare it to common arthropod reference genomes. The workflow we described is freely and publicly available via a web interface on CyVerse or as biocontainers that can be deployed scalably on local computing systems.

**Abstract:**

Genome sequencing of a diverse array of arthropod genomes is already underway, and these genomes will be used to study human health, agriculture, biodiversity, and ecology. These new genomes are intended to serve as community resources and provide the foundational information required to apply ‘omics technologies to a more diverse set of species. However, biologists require genome annotation to use these genomes and derive a better understanding of complex biological systems. Genome annotation incorporates two related, but distinct, processes: Demarcating genes and other elements present in genome sequences (structural annotation); and associating a function with genetic elements (functional annotation). While there are well-established and freely available workflows for structural annotation of gene identification in newly assembled genomes, workflows for providing the functional annotation required to support functional genomics studies are less well understood. Genome-scale functional annotation is required for functional modeling (enrichment, networks, etc.). A first-pass genome-wide functional annotation effort can rapidly identify under-represented gene sets for focused community annotation efforts. We present an open-source, open access, and containerized pipeline for genome-scale functional annotation of insect proteomes and apply it to various arthropod species. We show that the performance of the predictions is consistent across a set of arthropod genomes with varying assembly and annotation quality.

## 1. Introduction

Over the past decade, rapid developments of sequencing technologies and assembly tools and algorithms have moved the bottleneck in genomics from data generation to inference of biological function. Model organism databases with sustained manual curation efforts have provided a source for homology [1,2], and more recently, phylogeny-based [3] functional prediction for newly annotated gene sets. As we expand the sequencing efforts to organisms in hitherto poorly sampled branches of the eukaryotic tree of life [4], there is an increase in the number of novel proteins of unknown function, and even identifying genes closely related to previously studied genes in other species can be problematic. While workflows have been developed to support genome assembly and gene identification, the process for understanding the function of resulting gene products is not as well documented.

Annotation spans two related, but distinct, processes in genomics: Demarcating genes and other elements present in genome sequences (structural annotation); and associating a function with genetic elements (functional annotation). Here, we focus on functional annotation of gene sets based on Gene Ontology (GO) terms and metabolic pathways. Genome-scale functional annotation is required for functional modeling (enrichment, networks, etc.), and a first-pass genome-wide functional annotation effort can rapidly identify under-represented gene sets for focused community annotation efforts.

**Figure 1:**
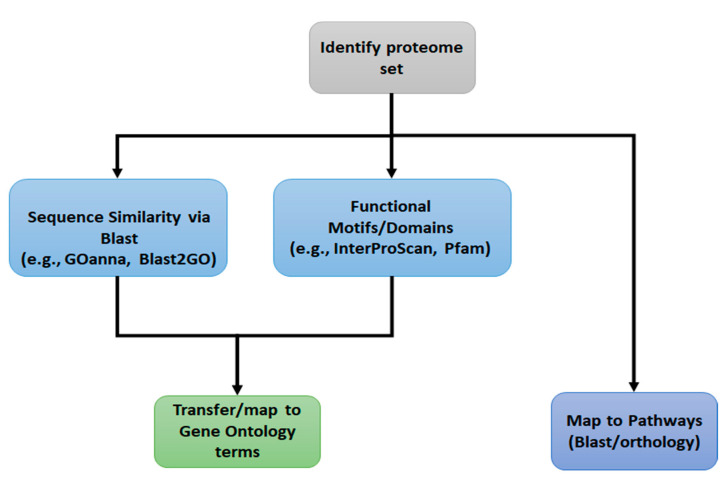
Generalized functional annotation workflow. The general approach for functional annotation is to combine GO annotations transferred based on sequence homology (e.g., BLAST) with information about functional motifs (e.g., derived from resources, such as PFAM). Gene products are mapped to metabolic and signaling pathways based upon sequence homology or orthology.

High throughput functional annotation relies on transferring functional information to unannotated proteins based upon analysis of functional domains and sequence homology [5,6]. While different software packages have been applied to this process, the general approach to first-pass functional annotation is similar (Figure 1). Protein sets are scanned for motifs and domains using resources like Pfam [7] and InterPro [8,9], and mapped to Gene Ontology terms using GO supplied mapping files. In addition to identifying shorter motifs and domains, BLAST analysis of full-length sequences can identify similar sequences which already have GO or pathway annotations linked to them. Examples of tools that rely on sequence similarity include GOanna [5], BLASTKoala [10], and Blast2GO [11]. More recently, the GO Consortium started using phylogenetic relationships to transfer GO terms [3]. The advantage of this approach is that evolutionary relationships provide more reliable evidence for conserved function than sequence similarity; however, this approach still relies on manual curation, which cannot keep pace with gene discovery from large-scale genome sequencing projects. Each of these sequence-based approaches relies on transferring GO terms associated with a gene product in one species to a gene product in another species, and the best practice for transferring GO terms is to limit this process to GO terms assigned based upon direct evidence [12].

### Motivation

Many high-quality arthropod genomes are being generated, in particular by large-scale genome projects, such as the USDA—Agricultural Research Service’s Ag100Pest Initiative [13,14], and others under the Earth BioGenome Project umbrella [15]. These new genomes serve as community resources and provide the foundational information required to apply ‘omics technologies to a more diverse set of species. Genome assemblies need structural and functional annotations to ensure that these ‘omics approaches can be rapidly translated into biological information that provides a better understanding of the system being studied. The Gene Ontology Consortium [16], UniProtKB [17], and KEGG [17,18] resources generate and maintain functional annotations of many proteomes available in the sequence databases, such as RefSeq and INSDC, and functional annotations produced by these initiatives are widely used and referenced by the scientific community. However, there is a delay before new genomes are processed by these databases, which have been exacerbated by the influx of new genome submissions. In addition, the process of manual curation of published papers is laborious and time-consuming for model species where most publications are focused on gene function [19]. A rapid, first-pass functional annotation workflow quickly provides functional information to support genomic analyses and experimentation and ensures that ‘omics approaches can be interpreted to better understand a diverse range of biological systems.

AgBase [20] and the i5k Workspace@NAL [21] databases serve the arthropod genomics community by providing access and curation tools for arthropod proteomes and genomes, respectively. Here, we report the creation of containerized workflows to fill the need for high-throughput functional annotation of proteins from eukaryotic genome sequencing programs for the scientific communities that we support, as well as the arthropod genomics community at large. We test these workflows using twelve sequenced invertebrate genomes selected to span a broad range of invertebrate classes and to represent genomes with varying assembly quality and sequencing technologies used. The proteins from these sequenced genomes are compared with three reference species, *Drosophila melanogaster*, *Apis mellifera* (honeybee), and *Tribolium castaneum* (red flour beetle), have well-characterized GO annotation based on experimental evidence. These workflows are also available on CyVerse to facilitate re-use [22,23] via a user-friendly web-based interface.

## 2. Materials and Methods

Complete instructions for running each component of the functional annotation pipeline on the command line, a high-performance computing cluster, or the CyVerse Discovery Environment can be found at the readthedocs site [24]. The specific tools used in this workflow are introduced below.

### 2.1. Sequence Similarity via BLAST: GOanna

GOanna [5] assigns GO terms based on sequence homology to specialized BLAST databases. These databases consist of proteins associated with GO terms, and grouped by phyla or taxonomic divisions (Table 1). GO uses several types of evidence to associate a GO term with a gene product: Direct experimental evidence, phylogenetic relatedness, and computational analysis. The established best practice for transferring GO terms between similar sequences is to only transfer GO terms based upon experimental evidence codes. This avoids making an inference based upon another inference, which could assign functions inappropriate to the organism’s physiology. GOanna accepts a protein FASTA file as input and allows the users to set standard BLAST parameters (Table S1). Since GOanna outputs results as a gene association file (GAF) file, it also requires users to provide information about the sequence source and species. Other information, such as protein name, is parsed from the FASTA header, and to ensure that it is correctly parsed from FASTA files generated by NCBI, an option to parse delimited sequence identifiers is also provided.

### 2.2. Functional Motif Analysis: InterProScan

InterPro ([8,9] is a database that integrates predictive information about protein function from a number of partner resources in the InterPro consortium. InterProScan [8,9] is a software tool that accepts a FASTA file, identifies motifs and domains from InterPro protein databases (Table 2), and maps them to GO terms and pathways with a number of customizable parameters (Table S2). Our containerized implementation also performs checks to trim any unknown amino acids at the end of sequences, including X’s, because the inclusion of these often causes the platform to fail. It also removes the “*” symbol added by some translation software to denote a stop codon before running submitted protein sequences in parallel. Parallelization is an important consideration for the scalability and utilization of high-performance computing resources. For nucleotide sequences, documentation is provided for using TransDecoder [25] to translate open reading frames from transcripts. Moreover, many other options for translating sequences into proteins are also publicly available. The XML output from InterProScan is parsed to produce the output GAF file and report pathway information.

### 2.3. Combining and QC of GO Annotations

The GOanna and InterProscan containers both output a GAF, the standard file format for GO annotation data. This is a tab-separated file that can be easily combined, but for use cases with large files that cannot be easily manipulated, we provide the Combine GAFs tool, which accepts multiple GAF files and combines them. It is possible to remove identical GO terms associated with the same protein by different software; but since these GO terms are assigned by different methods and have different evidence codes, we do not remove these at this step.

In addition to combining GAF files, the GO annotation data can be assessed using the GO Annotation Quality (GAQ) Score [19]. GAQ is a quantitative measure of the quality of GO annotation of a set of proteins. GAQ scores include the breadth of GO annotation, the level of detail of annotation, and the type of evidence used to infer the annotation. The scores generated can also be used to track changes in GO annotations over time. The GAQ tool determines the depth of each GO term and the rank of each evidence code associated with the annotation and returns a GAQ score as a product of depth and evidence code rank. The total GAQ score of each annotated gene product is calculated, and a summary is generated showing the overall total GAQ scores, the number of gene products annotated, and the average (mean) GAQ score of the whole protein set. We use the GAQ score to determine the value added to functional information, particularly when compared with well-annotated model species, such as *D. melanogaster*, and to a lesser extent, *Apis mellifera* and *Triboleum castaneum*.

### 2.4. Map to Pathways: KOBAS

KEGG Orthology Based Annotation System (KOBAS) [26,27] assigns input proteins to known pathways in KEGG. It also includes a gene set enrichment function (Table S3) to find statistically enriched genes in a disease or experimental condition with respect to the background of all annotated proteins in the organism. The pipeline consists of two modules:

- Annotate: This step assigns appropriate KEGG Ortholog (KO) terms for queried sequences based on a similarity search. It also assigns proteins to pathways from KEGG, Reactome, and BioCyc.
- Identify: This performs an enrichment analysis compared to a background of the species’ gene set among the annotation results based on the frequency or statistical significance of pathways.

For annotating the gene products from a species, we use the Annotate module.

### 2.5. Research Design and Method: Comparing Functional Annotation across Multiple Species

To test the usefulness of the functional annotation workflows, we selected a set of arthropod genomes (Table 3) with varying assembly quality and state of manual curation. This data set included several well-studied arthropod genomes, such as *Drosophila melanogaster*, *Apis mellifera*, and *Tribolium castaneum*, for comparison. BUSCO [26,28] version 5.1.2 was used with the protein option and arthropoda_odb10.2019-11-20 database with 1013 markers to analyze all protein sets for completeness (Table 4).

Proteome sets for each species were downloaded from NCBI and functionally annotated using the workflow described above. For GOanna, we used the invertebrate reference databases, and only the GO terms with experimental evidence were assigned (-b). Custom BLAST parameters included a BLAST identity (-g) and query coverage (-q) cutoff of 70% with a maximum number of gap opening size (-k) of 9 to account for insertion or deletion of short peptides. Ideally, the query and BLAST match should be of identical length, but we allowed for some flexibility (-r 1.2) to account for natural diversity and potential assembly or annotation errors. InterProScan was run to identify InterPro domains, GO terms, and pathways for the input proteins (-g -l -p -c), and we used all the databases to extract the maximum amount of information possible. A single, comprehensive GAF was obtained by combining the results from GOanna and InterProScan. The same protein sets were then run through KOBAS [26] to annotate pathways. The KOBAS Annotate tool (-a) used the *D. melanogaster* reference proteins (-s dme). The input data type must be specified (-t fasta:pro).

## 3. Results

### 3.1. Installation and Runtime Considerations

The memory usage and runtime of the containers, described here, scales with the size of the protein set except for InterProScan. A large number of databases (Table 2) that must be searched for matches for each protein sequence increases the runtime and memory usage for even small data sets. The scalability of InterProScan has been improved with data and compute parallelization. The input proteins are split into sets of 1000 sequences for parallel processing, but the time required for loading and searching all 16 databases is still significant. Another factor to consider is the increasing size of databases; new updates will only increase these requirements in the future. Therefore, we recommend that the InterProScan container be run on a high-performance computer like a cluster or a server with at least 256Gb of RAM and 500Gb of disk space. The documentation for this workflow [44] includes instructions on executing the containers with Singularity if Docker containers are not permitted, due to security restrictions. The GOanna and KOBAS containers can be set up on desktop-grade computers.

### 3.2. Parameter Optimization

Like all workflows, parameter optimization is a key part of ensuring quality results. Here we discuss the parameter optimization process for this workflow across a diverse range of arthropod genomes to consider when applying this workflow to their own data sets. For the GOanna tool, the key optimization parameters are the selection of the database and the standard BLAST parameters. It is common practice to do an initial BLAST search against a comprehensive database (e.g., NCBI nr or UniProt-SwissProt databases) to identify the most similar known sequence. While we include the UniProt SwissProt and TrEMBL database as options for GOanna, we note that the databases GOanna uses are not meant to be comprehensive, but rather a subset of proteins that have been assigned GO terms. Moreover, given that searching against larger databases increases the probability of finding spurious matches, we recommend using the phyla-specific database most relevant for your dataset and supplementing the output of GOanna matches with InterProScan results. To ensure high-quality results, BLAST parameters should be optimized. While many analyses report optimizing BLAST solely on the E-value, this varies based upon database size. To determine BLAST parameters, we randomly selected three sets of 1000 sequences from each of the proteomes and manually reviewed the results of alignments from BLAST run with default parameters. The most common error when these sets were re-run with more stringent E-values was identifying short, perfect matches (E-value = 0) that had low query coverage (e.g., less than 50%). To consistently return good matches from a broad range of protein sequences from all the proteomes used in this study, we used cutoffs of 70% identity and 70% coverage for the BLAST parameters.

Unlike GOanna, which is BLAST-based, InterProScan searches for near-perfect matches to short motifs and domains [9]. A key consideration for running InterProScan is to decide which databases should be searched. CDD or PFAM are frequently used, and both of these databases are included in the InterPro analysis. Since the computing requirements of InterProScan are considerable, these requirements could be reduced by searching fewer databases. While our workflow is deliberately designed to accept proteins, InterProScan can accept nucleotide sequences and translate them prior to searching the protein databases. Our initial tests indicated that submitting nucleotide sequences to InterProScan resulted in many more motif matches, but similar GO annotations (results not shown). Closer inspection revealed that the translation step produced large numbers of peptides, but many did not match the known peptides produced from the mRNA sequence used as input. Therefore, we recommend a separate translation step and submitting protein sequences to InterProScan.

To rapidly provide pathway annotations for arthropod gene products, we utilized the KEGG system, which maps genes to pathways based upon sequence homology, creating KEGG Ortholog (KO) sets for different species. Since the KOBAS annotate tool takes a sequence file and uses BLAST to associate KEGG pathways with these sequences, parameter optimization requires the selection of the database to search against (e.g., “KO” for all orthologous proteins or “dme” to restrict to only *D. melanogaster* proteins), as well as standard BLAST parameters. The parameters (-e -r -C -z, designated by * in Table S1) denoting E-value, rank, subject coverage, and orthologs for cross-species annotation can be modified to increase stringency when transferring annotation from the selected model species (-s). We note that the BLAST parameters required for this process may differ from GOanna because the two BLAST-related tools use different search databases.

### 3.3. Results and Discussion

#### 3.3.1. Genome Assembly

To test our functional annotation workflow, we selected twelve arthropod genomes, four of which were community-curated. The genomes were selected to represent a range of assembly quality and a diverse set of arthropod species. These twelve genomes were supplemented with three well-studied arthropods (a reference set): *Drosophila melanogaster* (fruit fly), *Apis mellifera* (honeybee), and *Tribolium castaneum* (red flour beetle) from the Orders Diptera, Hymenoptera, and Coleoptera, respectively. We note that all these species have been assembled, annotated, and the proteomes are considered mostly complete with BUSCO completeness scores ranging from 31 to 99% (Table 4). The genome assemblies for the selected species varied in contiguity and quality, with scaffold N50s ranging from 13.8 kb to 58.5 Mb (Table 3). Another metric of interest for quantifying the quality of the assembly before scaffolding is contig N50 that ranged from as low as 2.2 kb for genomes assembled with Illumina paired-end and mate-pair reads to 749.5 kb for genomes assembled with PacBio long-read technology (Table 3). Please note that assemblies with low contig N50, but comparatively high scaffold N50 can have large gaps filled with unknown (N) nucleotides.

The proteome sets we used ranged from 12,318–33,019 proteins (Table 3). We examined the proportion of these proteins that were annotated with GO data, and were also interested in determining what BLAST-based analyses contributed to this GO annotation compared to the motif-based InterProScan annotation. Overall, GO annotation ranged from 30–60% of the protein set, with an average of 45%, including the reference genomes. Notably, other species achieved the same rates of GO annotation as the reference gene sets, indicating that the workflow performs as expected. We also wanted to evaluate if assembly contiguity (contig and scaffold N50) and gene space completeness corresponded to coverage of functional annotation for the proteome. This was not always the case as 44.6% of the proteins from *L. lunatus* (caddisfly) were associated with GO terms, but the assembly only has a scaffold N50 of 54.6 kb and a contig N50 of 2.1 kb. The gene space for caddisfly is relatively incomplete at 42.4 with low duplication (Figure S1 and Table 4). On the other end of the spectrum, the hymenopteran *C. floridanum* (parasitoid wasp) has a contig and scaffold N50 of 14.5 kb and 1 Mb, respectively, but only 34.1% of its proteins must GO terms associated with them. The other hymenopteran in the test set, *A. rosae* (turnip sawfly), has a better GO term coverage of 57.05%, but it also has a more contiguous genome with a contig and scaffold N50 of 51.4 kb and 1 Mb, respectively. Both *A. rosae* (turnip sawfly) and *C. floridanum* (parasitoid wasp) have comparable BUSCO completeness metrics (99.7% and 93.7%), but duplication in the gene space is higher at 30.8% in *A. rosae* compared to only 1.2% in *C. floridanum*. It should be noted that highly curated reference genomes like *Drosophila melanogaster* have multiple isoforms annotated per protein (46.6 of 99.9) that are reported as duplicates by BUSCO.

#### 3.3.2. Gene Ontology Annotation

While metrics for assessing genome assembly and annotation are well-established, less work has been done on determining metrics for functional annotation. We measured the value of the GO terms assigned to gene products using the GO Annotation Quality (GAQ) Score [9,19]. The GAQ Score incorporates the breadth of annotation, the depth (or detail) of assigned GO terms, and the evidence for these assertions [9,19] to provide a quantitative score. A limitation of the GAQ Score is that it is relative and is best interpreted by determining improvements in the functional annotation of the same gene set over time with increasing GO annotation. To address this limitation of the GAQ Score, we provide the GAQ Score for *D. melanogaster* to compare a well-annotated reference gene set.

BLAST-based GO annotation assigned markedly fewer GO terms (accounting for at most only 4.09% of assigned annotations in caddisfly) (Table 5). However, the value of the GO annotations added by BLAST-based tools like GOanna is disproportional to the quantity of GO added by these tools. The average GAQ score for GO terms assigned by BLAST using GOanna was 142.02, while the average GAQ score of GO terms assigned by InterProScan based on motif search was 34.84. The GAQ score of the *D. melanogaster* functional annotation downloaded from the European Bioinformatics Institute (EBI) [45], which included manual annotation, had a much higher GAQ score of 243.68 as it included evidence codes for manual functional annotation, which are weighted higher than sequence similarity-based GO term assignment.

In addition to measuring how the assembly quality and proteome completeness influenced the GO term annotation, another question of interest was the potential influence of the phylogenetic distance from the model species, specifically *Drosophila melanogaster*. Among the reference genomes, *D. melanogaster* is by far the best annotated and curated. Since GOanna uses a database of experimentally validated GO terms wherein *D. melanogaster* was the model system used, 14.8% of *D. melanogaster* proteins were annotated with GO terms by GOanna compared to 2.5% and 2.6% for the honeybee and red flour beetle, respectively (Table 5).

Both *D. citri* (Asian citrus psyllid) and *V. destructor* (parasitic mite) showed overall annotation comparable to the selected references making the case that good quality genomes and annotation provide the best foundation for successful functional annotation. Surprisingly, the hymenopteran *A. rosae* (turnip sawfly) with a 99.7 BUSCO completeness, but lower contig N50 (51.4 kb) and scaffold N50 (1.3 Mb) than *D. citri* and *V. destructor* also fared well for overall annotation. The contiguous *D. citri* and *V. destructor* genomes did not have the highest BUSCO completeness scores (87.1% and 95.9%). The BUSCO ortholog set is computed based on a set of conserved genes in a clade, and the hemipteran clade is relatively under-sampled among arthropods, so this score might change in the future as more hemipteran genomes are sequenced.

*C. capitata* (Mediterranean fruit fly) had the highest percentage of proteins annotated by GOanna (7.9%), but that is somewhat expected considering its phylogenetic closeness to the reference species, *D. melanogaster*. The *L. lunatus* (caddisfly) and *L. hesperus* (Western black widow spider) genomes have the lowest contig N50, scaffold N50 metrics, and BUSCO completeness scores, but 44.6% of *L. lunatus* proteins were annotated compared to 31.17% of *L. hesperus* proteins. *E. affinis* (calanoid copepod) scored the poorest on GO annotation among our test species with only 30% of proteins annotated, possibly due to its phylogenetic distance from *D. melanogaster*, despite having a better contig and scaffold N50 of 5.7 kb and 862.6 kb, respectively. However, it had a poor BUSCO completeness metric with only 57.5% completeness and 22.5% missing orthologs. We found a common theme in our test set and related analysis, whereby the quality and depth of functional annotation were inversely proportional to the phylogenetic distance from the *D. melanogaster* model species (data not shown).

There are two major approaches for associating GO with gene products—manual curation of published literature on gene function and sequence analysis. The former approach has been limited to less than 20 species, and it is unlikely that most species across the kingdom of life will get the benefit of the sustained manual curation based on experimental evidence from published literature. In response, GO curators have developed a method for transferring GO functions across species based upon phylogenetic analysis [3], but this method still relies on manual review by expert curators. While our GO annotation workflow provides a rapid method to associate GO function with proteomes, our results emphasize the need for the better annotation of non-model species in every major clade so that proteins from newly sequenced genomes can be assigned function more accurately. One approach to achieve this goal would be to combine the GO phylogenetic approach to identify genes not found in *D. melanogaster* and identify invertebrate species with existing functional literature for targeted GO curation by providing more direct evidence annotations for invertebrate gene products. This focused application of phylogenetics and targeted manual curation would likely provide GO annotation that would impact the annotation of invertebrate gene function. We note that this is also a limitation of our sequence-based approach as it is dependent on manual biocuration of published papers from reference species (e.g., *D. melanogaster*) to provide a useful level of functional detail. Another limitation of our workflow is that it is currently limited to proteins and does not provide information about ncRNA function.

#### 3.3.3. Pathway Annotation

High throughput sequencing has enabled the profiling of longitudinal transcriptional response at the organismal, tissue, and single-cell level in addition to multiple life stages and conditions. Although GO terms are highly effective at deducing the changes in gene expression, pathway-level perturbations provide valuable biological insight for the interpretation of functional genomics data sets and are critical for integrating proteome and metabolome data sets to understand phenotypes. Therefore, we were also interested in automatically reconstruct metabolic pathways from the proteomes from a range of arthropod genomes.

Pathways data is provided by resources, such as KEGG [46], Reactome [47], and BioCyc [48], and as we developed our workflow, we selected KEGG pathways for our workflow because it supports the most extensive set of invertebrate species, and the KOBAS tool is freely available [26]. In our initial tests using the KOBAS tool to annotate pathways, we determined that comparing the arthropod proteome sets against the KEGG *Drosophila melanogaster* (‘dme’) provide the most comprehensive results, and this well-studied arthropod species also has the broadest set of functional information based on experimental validation, including pathways.

Not surprisingly, *A. mellifera* and *T. castaneum* references had similar proportions of proteins assigned to pathways, although a slightly lower number of proteins per pathway than *D. melanogaster* (Table 6). The reference species had about one-third of proteins assigned to pathways, and most of the test species were annotated to the same degree or better. Curiously, several species did substantially better than the reference set: *V. destructor*, *A. rosae*, *D. citri*, and *L. lunatus* all had about 40% of proteins assigned to pathways, and a similar effect was seen for the GO annotation in these species. We note that most of these species have well-assembled genomes with high contig and scaffold N50 and BUSCO completeness scores. The average number of proteins per pathway scaled with the genome contiguity and BUSCO duplication rate, suggesting that the higher gene copy number accounts for this variance (Figures S1 and S2).

## 4. Conclusions

Our results with a test set of arthropod genomes that are phylogenetically divergent and at different levels of assembly and annotation quality demonstrate the overall utility of our workflow to rapidly provide functional annotation for proteins. We are currently working on expanding functional annotation to include noncoding RNAs. Our workflow assigns GO and pathways information to 40–60% of proteins. While starting with a contiguous chromosomal length genome assembly and an evidence-based protein set is ideal, we expect that species with complete gene models are sufficient to get a first-pass functional annotation. This functional information can be of immediate use to the community to support functional and comparative studies, including those generated by the Ag100Pest Initiative and other genomes hosted by the i5k Workspace@NAL. However, we would like to caution the user that the data sets underlying any functional annotation workflows are continually changing, and any functional annotation set should be refreshed periodically irrespective of whether the genome sequencing and annotation have changed. Furthermore, functional annotation provides information about pathways and gene families that are poorly annotated or absent from gene sets, providing useful information that can be used to direct targeted manual curation of genes. Manual curation of gene models is a well-established activity in the arthropod research community using Apollo [49] through community databases, such as the i5k Workspace@NAL [21], VectorBase [50], the Hymenoptera Genome Database [51], Citrus Greening Database [52,53,54,55,56,57,58], and others. Functional annotation would support this focus while extending the utility of the genome for the research community.

## Figures and Tables

**Table 1:** GOanna version 2.2 databases. Databases are prepared from proteins that have GO annotations based upon taxonomic divisions. Protein numbers reported as of January 2019.

Database Name	No. UniProtKB Proteins	No. in GOanna Db
arthropod	3,956,843	12,081
bacteria	28,660,834	12,748
bird	777,091	1379
fish	1,505,807	12,478
fungi	7,614,812	13,718
human	161,566	21,125
insecta	2,883,005	11,886
invertebrates	8,409,505	20,741
mammals	1,836,549	42,966
nematode	1,541,602	4941
plants	6,300,920	16,058
UniProt-SwissProt	50,258	72,337
UniProt-TrEMBL	4,720,107	57,834

**Table 2:** Databases used by InterProScan version 5.45-80 for annotation.

Database	Description
TIGRFAM	TIGRFAMs are protein families based on Hidden Markov Models or HMMs.
SFLD	SFLDs are protein families based on Hidden Markov Models or HMMs.
ProDom	ProDom is a comprehensive set of protein domain families automatically generated from the UniProt Knowledge Database.
Hamap	High-quality Automated and Manual Annotation of Microbial Proteomes.
SMART	SMART identifies and analyzes domain architectures based on Hidden Markov Models or HMMs.
CDD	Prediction of CDD domains in proteins.
ProSiteProfiles	PROSITE consists of documentation entries describing protein domains, families, and functional sites, as well as associated patterns and profiles to identify them.
ProSitePatterns	PROSITE consists of documentation entries describing protein domains, families, and functional sites, as well as associated patterns and profiles to identify them.
SUPERFAMILY	SUPERFAMILY is a database of structural and functional annotation for all proteins and genomes.
PRINTS	A fingerprint is a group of conserved motifs used to characterize a protein family.
PANTHER	The PANTHER (protein analysis through evolutionary relationships) Classification System is a unique resource that classifies genes by their functions, using published scientific experimental evidence and evolutionary relationships to predict function even in the absence of direct experimental evidence.
Gene3D	Structural assignment for whole genes and genomes using the CATH domain structure database.
PIRSF	The PIRSF concept is being used as a guiding principle to provide comprehensive and non-overlapping clustering of UniProtKB sequences into a hierarchical order to reflect their evolutionary relationships.
Pfam	A large collection of protein families, each represented by multiple sequence alignments and hidden Markov models (HMMs).
Coils	Prediction of Coiled Coil Regions in proteins.
MobiDBLite	Prediction of disordered domains regions in proteins.

**Table 3:** Arthropod genomes selected for this study and their assembly and annotation statistics. The test species are sorted by the scaffold N50 value.

Species	Genome Assembly Accession	Genome Assembly Name	Contig N50	Scaffold N50	Annotation Name	Proteins	Proteins Assigned GO Terms	Source
*Apis mellifera* (honey bee)	GCA_000002195.1	Amel_4.5	5,832,476	13,619,445	OGSv3.3	15,314	39.91%	[29]
*Drosophila melanogaster* (fruit fly)	GCA_000001215.4	DMEL_r6.36	21,485,538	25,286,936	FB2020_05	30,724	59.42%	[30]
*Tribolium castaneum* (red flour beetle)	GCA_000002335.3	TCAS_5.2	73,049	4,456,720	TCAS_OGS_v3	18,534	44.98%	[31]
*Latrodectus hesperus* (Western black widow spider)	GCA_000697925.1	Lhes_1.0	2223	13,889	LHES-BCM_version_0.5.3	17,364	31.17%	[32]
*Limnephilus lunatus* (caddisfly)	GCA_000648945.1	Llun_1.0	2103	54,650	LLUN-BCM_version_0.5.3	13,292	55.76%	[33]
*Oncopeltus fasciatus* (large milkweed bug)	GCA_000696205.1	Ofas_1.0	4047	339,960	oncfas_OGSv1.2	19,793	34.31%	[34]
*Homalodisca vitripennis* (glassy-winged sharpshooter)	GCA_000696855.1	Hvit_1.0	4857	512,049	HVIT-BCM_version_0.5.3	33,019	38.00%	[35]
*Eurytemora affinis* (calanoid copepod)	GCA_000591075.1	Eaff_1.0	5738	862,645	EAFF-BCM_version_0.5.3	29,783	30.02%	[36]
*Agrilus planipennis* (emerald ash borer)	GCA_000699045.1	Apla_1.0	6314	910,924	APLA-BCM_version_0.5.3	15,497	51.07%	[37]
*Copidosoma floridanum* (parasitoid wasp)	GCA_000648655.1	Cflo_1.0	14,521	1,037,125	CFLO-BCM_version_0.5.3	19,869	34.14%	[38]
*Athalia rosae* (turnip sawfly)	GCA_000344095.1	Aros_1.0	51,418	1,366,867	AROS-BCM_version_0.5.3	22,213	57.05%	[39]
*Ceratitis capitata* (Mediterranean fruit fly)	GCA_000347755.2	Ccap_1.1	45,879	4,118,346	Ccap-OGSv1	12,318	55.75%	[40]
*Cimex lectularius* (Cimicidae bed bug)	GCA_000648675.1	Clec_1.0	23,511	7,172,596	Clec-OGSv1.2	14,212	49.42%	[41]
*Varroa destructor* (parasitic mite)	GCA_002443255.1	Vdes_3.0	201,886	58,536,683	NCBI Varroa destructor Annotation Release 100	30,221	53.60%	[42]
*Diaphorina citri* (Asian citrus psyllid)	NA	Version 3	749,525	40,596,296	OGSv3	19,049	59.30%	[43]

**Table 4:** Arthropod genomes selected for this study and their BUSCO completeness statistics. The test species are sorted by the BUSCO completeness score. BUSCO version 5.1.2 was used with the protein option and arthropoda_odb10.2019-11-20 database with 1013 markers.

Species	Complete	Complete Single-Copy	Complete Duplicated	Fragmented	Missing
*Drosophila melanogaster* (fruit fly)	99.90	53.3	46.6	0	0.1
*Athalia rosae* (turnip sawfly)	99.70	68.9	30.8	0	0.3
*Ceratitis capitata* (Mediterranean fruit fly)	98.40	97.5	0.9	0.4	1.2
*Tribolium castaneum* (red flour beetle)	98.40	93.1	5.3	1.2	0.4
*Apis mellifera* (honey bee)	97.40	96.9	0.5	1.5	1.1
*Varroa destructor* (parasitic mite)	95.90	43.1	52.8	0.7	3.4
*Cimex lectularius* (Cimicidae bed bug)	95.30	93.5	1.8	2.5	2.2
*Copidosoma floridanum* (parasitoid wasp)	93.70	92.5	1.2	2.9	3.4
*Agrilus planipennis* (emerald ash borer)	90.90	89.1	1.8	4.6	4.5
*Diaphorina citri* (Asian citrus psyllid)	87.10	55.9	31.2	2.8	10.1
*Oncopeltus fasciatus* (large milkweed bug)	72.90	70.8	2.1	21.4	5.7
*Eurytemora affinis* (calanoid copepod)	57.50	55.9	1.6	20	22.5
*Homalodisca vitripennis* (glassy-winged sharpshooter)	55.90	54.2	1.7	32.5	11.6
*Limnephilus lunatus* (caddisfly)	42.40	41.4	1	28.1	29.5
*Latrodectus hesperus* (Western black widow spider)	31.40	30.6	0.8	26.9	41.7

**Table 5:** GOanna and InterProScan results for arthropod genomes selected for this study. The test species are sorted by their GO term coverage.

Species	Proteins	Proteins Assigned GO Terms	GOanna (BLAST)		InterProScan (Motif Analysis)	
			Proteins Assigned GO	Average GAQ	Proteins Assigned GO	Average GAQ
*Apis mellifera* (honey bee)	15,314	39.91%	2.59%	164.796	39.32%	33.745
*Drosophila melanogaster* (fruit fly)	30,724	59.42%	14.85%	142.024	53.12%	34.847
*Tribolium castaneum* (red flour beetle)	18,534	44.98%	2.64%	142.27	44.36%	33.585
*Diaphorina citri* (Asian citrus psyllid)	19,049	59.30%	2.23%	168.358	57.46%	34.44
*Athalia rosae* (turnip sawfly)	22,213	57.05%	2.11%	144.594	56.67%	35.317
*Varroa destructor* (parasitic mite)	30,221	53.60%	0.52%	167.385	53.53%	33.704
*Agrilus planipennis* (emerald ash borer)	15,497	51.07%	2.87%	179.869	41.27%	31.368
*Ceratitis capitata* (Mediterranean fruit fly)	14,212	49.42%	7.94%	127.988	46.42%	32.504
*Cimex lectularius* (Cimicidae bed bug)	14,212	49.26%	3.00%	177.746	48.33%	35.017
*Limnephilus lunatus* (caddisfly)	13,292	44.61%	4.09%	172.298	43.03%	31.353
*Homalodisca vitripennis* (glassy-winged sharpshooter)	33,019	38.00%	1.53%	174.869	30.22%	30.751
*Oncopeltus fasciatus* (large milkweed bug)	19,793	34.31%	2.73%	189.411	33.24%	29.997
*Copidosoma floridanum* (parasitoid wasp)	19,869	34.14%	1.98%	168.485	33.63%	31.466
*Latrodectus hesperus* (Western black widow spider)	17,364	31.17%	2.02%	197.44	30.44%	28.896
*Eurytemora affinis* (calanoid copepod)	29,783	30.02%	0.71%	157.137	23.58%	30.221

**Table 6:** KOBAS results for arthropod genomes selected for this study. The test species are sorted by the overall proportion of proteins assigned to pathways.

		All Pathways		KEGG Pathways	
Species	Proteins	Proteins Assigned to Pathways	Average Number of Proteins in Pathways	% Assigned to Pathways	Average Number of Proteins in Pathways
*Apis mellifera* (honeybee)	15,314	29.27%	3.41	17.57%	20.23
*Drosophila melanogaster* (fruit fly)	30,724	37.73%	8.77	21.24%	49.08
*Tribolium castaneum* (red flour beetle)	18,534	30.03%	4.22	16.99%	23.68
*Varroa destructor* (parasitic mite)	30,221	41.55%	9.63	23.50%	54.62
*Athalia rosae* (turnip sawfly)	22,213	40.95%	6.9	22.79%	38.06
*Diaphorina citri* (Asian citrus psyllid)	19,049	40.07%	5.88	23.72%	34.75
*Limnephilus lunatus* (caddisfly)	13,292	38.09%	3.92	22.94%	23.10
*Cimex lectularius* (Cimicidae bed bug)	14,212	37.07%	4.01	22.50%	24.22
*Ceratitis capitata* (Mediterranean fruit fly)	12,318	35.91%	3.35	21.36%	19.78
*Oncopeltus fasciatus* (large milkweed bug)	19,793	32.51%	4.9	18.36%	27.53
*Agrilus planipennis* (emerald ash borer)	15,497	31.81%	3.74	18.92%	22.05
*Latrodectus hesperus* (Western black widow spider)	17,364	30.06%	4.06	16.97%	22.66
*Homalodisca vitripennis* (glassy-winged sharpshooter)	33,019	25.41%	6.39	15.06%	37.68
*Copidosoma floridanum* (parasitoid wasp)	19,869	25.35%	3.83	14.43%	21.56
*Eurytemora affinis* (calanoid copepod)	29,783	20.55%	4.69	11.42%	25.58

## Data Availability

The outputs from the workflow for each genome will be made available on AgData Commons. The docker containers are available at docker hub: GOanna [59], InterProScan [60], Combine GAFs [61], and KOBAS [62]. The source code for constructing the GOanna, InterProScan, Combine GAF, and KOBAS containers is available on GitHub [63,64,65,66,67].

## Supplementary Materials

The following are available online at https://www.mdpi.com/article/10.3390/insects12080748/s1, Figure S1: Duplication in BUSCO single-copy orthologs: Plot of duplication (%) of 1013 single-copy orthologs against the scaffold N50 showing correlation of increasing duplication with an increase in contiguity of the assembly, Figure S2: Average number of proteins per pathway: Plot of the average number of proteins per pathway against the scaffold N50 showing a correlation of increasing protein count with an increase in contiguity of the assembly, Table S1. GOanna version 2.2 parameters. Parameters are mainly based upon standard BLAST parameters and are categorized into required and optional. The parameters recommended for optimization are denoted with an *, Table S2. InterProScan version 5.45-80 parameters. The parameters are categorized into required and optional. The parameters recommended for optimization are denoted with an *, Table S3. KOBAS version 3.0.3 parameters. The parameters are categorized into required and optional. The parameters recommended for optimization are denoted with an *.
