# Supplementary material for: Workflows for Rapid Functional Annotation of Diverse Arthropod Genomes
Source: Insects. 2021 Aug 19;12(8):748. doi: 10.3390/insects12080748 (PMC8397112; doi:10.3390/insects12080748)
Supplement: Supplementary file 1 [file insects-12-00748-s001.zip › insects-1281406-supplementary.pdf]

insects

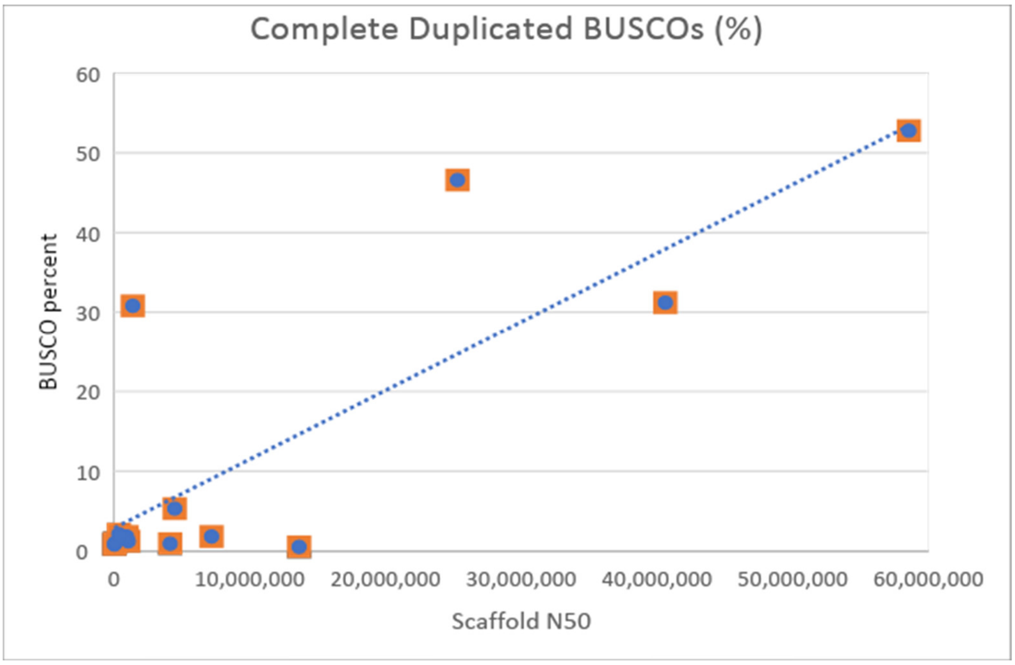

Figure S1. Duplication in BUSCO single-copy orthologs: Plot of duplication (%) of 1013 single-copy orthologs against the scaffold N50 showing correlation of increasing duplication with an increase in contiguity of the assembly.

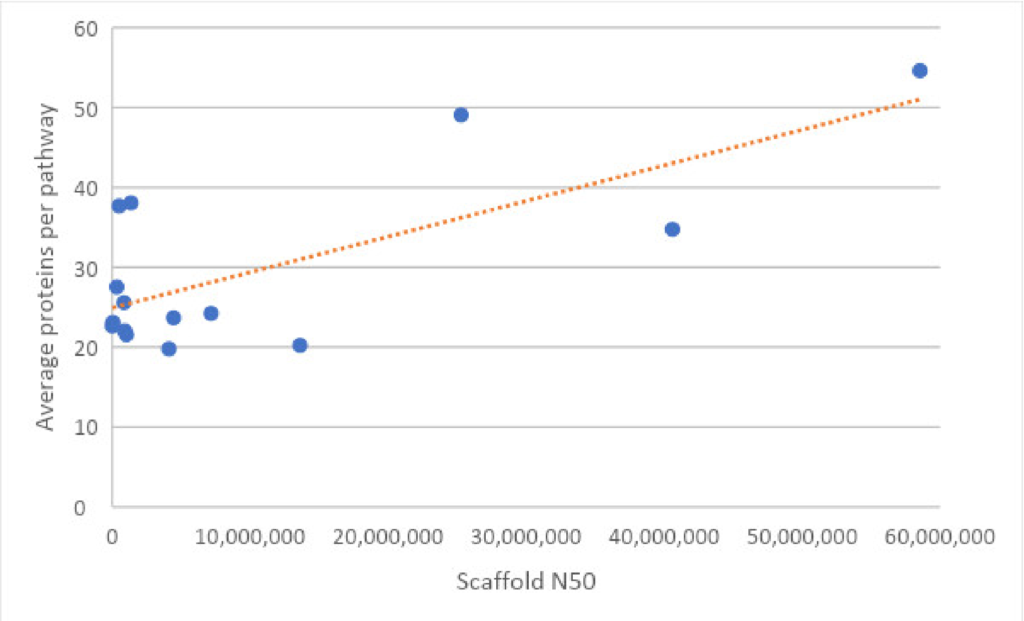

Figure S2. Average number of proteins per pathway: Plot of the average number of proteins per pathway against the scaffold N50 showing a correlation of increasing protein count with an increase in contiguity of the assembly.

Table S1. GOanna version 2.2 parameters. Parameters are mainly based upon standard BLAST parameters and are categorized into required and optional. The parameters recommended for optimization are denoted with an *.

Option	Description
	Required parameters
-a *	BLAST database basename ('arthropod', 'bacteria', 'bird', 'crustacean', 'fish', 'fungi', 'human', 'insecta','invertebrates', 'mammals', 'nematode', 'plants', 'rodents' 'uniprot_sprot', 'uniprot_trembl', 'vertebrates' or 'viruses')
-c	Peptide FASTA filename
-o	BLAST output file basename
	Optional parameters
-b *	Transfer GO with experimental evidence only ('yes' or 'no'). Default = 'yes'.
-d	Database of query ID. If your entry contains spaces, either substitute and underscore (_), or, to preserve the space, use quotes around your entry. Default: 'user_input_db'
-e *	Expect value (E) for saving hits. Default is 10.
-f	Number of aligned sequences to keep. Default: 3
-g	BLAST percent identity above which match should be kept. Default: keep all matches.
-h	Help
-m *	BLAST percent positive identity above which match should be kept. Default: keep all matches.
-s	Bit score above which match should be kept. Default: keep all matches.
-k *	Maximum number of gap openings allowed for match to be kept. Default: 100
-l	Maximum number of total gaps allowed for match to be kept. Default: 1000
-q *	Minimum query coverage per subject for match to be kept. Default: keep all matches
-r *	Ratio of query length to subject length. Lengths should be comparable for matches to be kept. Default: less than 1.2, so a difference of up to 20% can be tolerated
-t	Number of threads. Default: 8
-u	'Assigned by' field of your GAF output file. If your entry contains spaces (e.g., firstname lastname) either substitute and underscore (_) or, to preserve the space, use quotes around your entry (e.g., firstname lastname) Default: 'user'
-x	Taxon ID of the peptides you are BLASTing. Default: 'taxon:0000′
-p	parse_deflines. Parse query and subject bar delimited sequence identifiers

Table S2. InterProScan version 5.45-80 parameters. The parameters are categorized into required and optional. The parameters recommended for optimization are denoted with an *.

Option	Description
	Required parameters
-i	path to FASTA file that should be loaded on Master startup. Alternatively, in CONVERT mode, the InterProScan 5 XML file to convert.
	Optional parameters
-a *	Comma separated list of analyses. If this option is not set, ALL analyses will be run.<br>Available analyses:<br>• TIGRFAM<br>• SFLD<br>• ProDom<br>• Hamap<br>• SMART<br>• CDD<br>• ProSiteProfiles<br>• ProSitePatterns<br>• SUPERFAMILY<br>• PRINTS<br>• PANTHER<br>• Gene3D<br>• Pfam<br>• Coils<br>• MobiDBLite
-b	Base output filename (relative or absolute path).<br>Note that this option, the output directory (-d) option, and the output file name (-o) option are mutually exclusive. The appropriate file extension for the output format(s) will be appended automatically. By default the input file path/name will be used.
-d	Output directory.<br>Note that this option, the output file name (-o) option, and the output file base (-b) option are mutually exclusive. The output filename(s) are the<br>same as the input filename, with the appropriate file extension(s) for the output format(s) appended automatically.
-c *	Disables the use of the precalculated match lookup service from EBI. All match calculations will be run locally.
-C	Supply the number of cpus to use.
-e	Excludes sites from the XML, JSON output
-f	Case-insensitive, comma separated list of output formats. Supported formats are TSV, XML, JSON, GFF3, HTML and SVG. Default for protein sequences are TSV, XML and GFF3, or for nucleotide sequences GFF3 and XML.
-g *	Switch on lookup of corresponding Gene Ontology annotation (IMPLIES -l lookup option)

-h	Display help information
-l	Also include lookup of corresponding InterPro annotation in the TSV and GFF3 output formats.
-m	Minimum nucleotide size of ORF to report. Will only be considered if n is specified as a sequence type. Please be aware of the fact that if you specify a too short value it might be that the analysis takes a very long time!
-o	Explicit output file name (relative or absolute path).<br>Note that this option, the output directory -d option, and the output file basename -b option are mutually exclusive. If this option is given, you MUST specify a single output format using the -f option. The output file name will not be modified. Note that specifying an output file name using this option OVERWRITES ANY EXISTING FILE.
-p *	Switch on lookup of corresponding Pathway annotation (IMPLIES -l lookup option)
-t	The type of the input sequences (dna/rna (n) or protein (p)). The default sequence type is protein.
-T	Specify temporary file directory (relative or absolute path). The default location is temp/.
-v	Display version number
-r	'Mode' required (-r 'cluster') to run in cluster mode. These options are provided but have not been tested with this wrapper script. For more information on running InterProScan in cluster mode [66]
-R	Cluster run id (crid) required when using cluster mode.
-F	This is the output directory from InterProScan.(XML parser option)
-D	Supply the database responsible for these annotations. (XML parser option)
-x	NCBI taxon ID of the ID being annotated (XML parser option)
-y	Transcript or protein (XML parser option)
-n	Name of the biocurator who made these annotations (XML parser option)
-M	Mapping file (XML parser option)
-B	Bad input sequence file (XML parser option)

Table S3. KOBAS version 3.0.3 parameters. The parameters are categorized into required and optional. The parameters recommended for optimization are denoted with an *.

Option	Description
	Required parameters
-i	INFILE can be FASTA or one-per-line identifiers. See -t intype for details.
-s *	SPECIES 3 or 4 letter species abbreviation (can be found here: 4ftp://ftp.cbi.pku.edu.cn/pub/KO-BAS_3.0_DOWNLOAD/species_abbr.txt or here:[67]
-o	OUTPUT file (Default is stdout.)
-t	INTYPE (fasta:pro, fasta:nuc, blastout:xml, blastout:tab, id:ncbigi, id:uniprot, id:ensembl, id:ncbigene), default fasta:pro
-a or -g	-a runs KOBAS Annotate and -g runs KOBAS Identify. One of these options has to be used. Otherwise -j can be used to run both
	Optional parameters
-l	LIST available species, or list available databases for a specific species
-e *	EVALUE expect threshold for BLAST, default 1e-5
-r *	RANK rank cutoff for valid hits from BLAST result, default is 5
-C *	COVERAGE subject coverage cutoff for BLAST, default 0
-z *	ORTHOLOG whether only use orthologs for cross-species annotation or not, default NO (if only using orthologs, please provide the species abbreviation of your input
-k	KOBAS HOME The path to kobas_home, which is the parent directory of sqlite3/and seq_pep/. This is the absolute path in the container.
-v	BLAST HOME The path to blast_home, which is the parent directory of blastx and blastp. This is the absolute path in the container.
-y	BLASTDB The path to seq_pep/. This is the absolute path in the container.
-q	KOBASDB The path to sqlite3/, This is the absolute path in the container.
-p	BLASTP The path to blastp. This is the absolute path in the container.
-x	BLASTX The path to blastx. This is the absolute path in the container.
-T	number of THREADS to use in BLAST search. Default = 8
-f	FGFILE foreground file, the output of annotate (KOBAS identify option)
-b	BGFILE background file, species abbreviation, see this list for species codes: [67] (KOBAS identify option)

-d	DB databases for selection, 1-letter abbreviation separated by/: K for KEGG PATHWAY, n for PID, b for BioCarta, R for Reactome, B for BioCyc, p for PANTHER, o for OMIM, k for KEGG DISEASE, f for FunDO, g for GAD, N for NHGRI GWAS Catalog and G for Gene Ontology, default K/n/b/R/B/p/o/k/f/g/N/(KOBAS identify option)
-m	METHOD choose statistical test method: b for binomial test, c for chi-square test, h for hypergeometric test/Fisher's exact test, and x for frequency list, default hypergeometric test/Fisher's exact test (KOBAS identify option)
-n	FDR choose false discovery rate (FDR) correction method: BH for Benjamini and Hochberg, BY for Benjamini and Yekutieli, QVALUE, and None, default BH (KOBAS identify option)
-c	CUTOFF terms with less than cutoff number of genes are not used for statistical tests, default 5 (KOBAS identify option)
